# Crosstalk Between the Gut and Brain: Importance of the Fecal Microbiota in Patient With Brain Tumors

**DOI:** 10.3389/fcimb.2022.881071

**Published:** 2022-06-17

**Authors:** Yuping Li, Haixiao Jiang, Xiaolin Wang, Xiaoguang Liu, Yujia Huang, Zhiyao Wang, Qiang Ma, Lun Dong, Yajie Qi, Hengzhu Zhang, Guangyu Lu

**Affiliations:** ^1^ Neuro Intensive Care Unit, Department of Neurosurgery, Clinical Medical College, Yangzhou University, Yangzhou, China; ^2^ Department of Neurosurgery, Yangzhou Clinical Medical College of Xuzhou Medical University, Xuzhou, China; ^3^ Department of Neurosurgery, Affiliated Hospital of Yangzhou University, Yangzhou, China; ^4^ Department of Thoracic Surgery, Northern Jiangsu People’s Hospital, Clinical Medical College, Yangzhou University, Yangzhou, China; ^5^ Department of Neurosurgery, Clinical Medical College, Yangzhou University, Yangzhou, China; ^6^ School of Public Health, Yangzhou University, Yangzhou, China

**Keywords:** gut microbiome, biomarker, microbiota-gut-immune, brain-gut axis, brain tumors

## Abstract

**Background:**

Variations in the gut microbiota may affect the metabolism, inflammation and immune response of the host. Microbiota dysbiosis has been extensively investigated in neurological disorders and diseases of the central nervous system (CNS). However, the alterations of the gut microbiota in patients suffering from brain tumors and the associations of the gut microbiota with these diseases remain unknown. Herein, we investigate the alterations of the gut microbiota community in patients with brain tumors and the associations between the two and further explore microbial markers used for the diagnosis of brain tumors.

**Methods:**

In our study, we recruited 158 participants, consisting of 101 brain tumor patients (65 benign and 36 malignant cases) and 57 age- and sex-matched healthy controls (HCs). We characterized the gut microbial community by using 16S rRNA gene amplicon sequencing and investigated its correlations with clinical features.

**Results:**

The results showed remarkably less microbial ecosystem richness and evenness in patients with brain tumors than in HCs. The gut microbiota community structure underwent profound changes in the brain tumor group, including an increase in the abundances of pathogenic bacteria, such as *Fusobacteriota* and *Proteobacteria* and a reduction in the abundances of probiotic bacteria, such as *Bifidobacterium* or *Lachnospira*. Moreover, our study indicated more significant correlations and clustering of pathogens in the malignant brain tumor group. Furthermore, a biomarker panel was used to discriminate the brain tumor patients from the healthy controls (AUC: 0.77). Kyoto Encyclopedia of Genes and Genomes (KEGG) annotation revealed an accumulation of harmful metabolites and disorders of the basic physiological pathways in the brain tumor group.

**Conclusions:**

Our study revealed that brain tumor patients may possess divergent host-microbe interactions from those of healthy controls, especially in malignant brain tumor patients. In addition, the intestinal flora may be involved in immune responses and metabolism in the microenvironment of brain tumors. All evidence, including the biomarker panel, suggests that the intestinal flora may be a useful diagnostic and predictive tool and an important preventive target for brain tumors.

## Background

The annual global age-standardized incidence and mortality for all primary tumors in the central nervous system (CNS) are approximately 23.41 and 4.42 per 100,000, respectively (Ostrom et al., 2019). Brain tumors can be divided into several types on the basis of the histological phenotypes, gene mutations and molecular profiles. The most common brain tumors are malignant gliomas and benign meningiomas, which account for 47.7% and 37.6% of all brain tumors, respectively (Ostrom et al., 2019). It is known that both genetic and mutation factors are associated with the etiology and development of various brain tumors (Barnholtz-Sloan et al., 2018; Ostrom et al., 2019). Mutations targeting isocitrate dehydrogenase 1/2 (IDH 1/2) have been demonstrated to be associated with characteristic DNA methylation patterns (Gusyatiner and Hegi, 2018). The loss of the neurofibromatosis type II gene could contribute to the formation of meningiomas and cranial nerve schwannomas (Coy et al., 2020). Recently, several studies have indicated that environmental and nongenetic endogenous factors are closely related to several diseases and neurological disorders in the CNS.

The human gut, one of the most dynamic niches, harbors trillions of microbiome constituents, including bacteria, fungi and other species (Milani et al., 2017). The microbiome plays an essential role in the maintenance of host homeostasis by regulating several important physiological processes, including nutrient metabolism, maturation, and stimulation of angiogenesis (Zmora et al., 2019). Moreover, accumulating evidence suggests that the structures and metabolites of the intestinal flora could be important environmental factors in several cerebral diseases through a bidirectional pathway termed the gut-brain axis (Rutsch et al., 2020; Morais et al., 2021). For example, the structural alteration of the gut microbiota in Alzheimer’s disease can stimulate the differentiation and proliferation of proinflammatory Th1 cells by the accumulation of isoleucine (Wang et al., 2019). α-Synuclein, the hallmark protein of Parkinson’s disease, can be generated by gut bacteria and transported to the brain *via* the vagus nerve (Cryan et al., 2020). Several studies have indicated that dysbiosis affecting the bidirectional communication of the gut-brain axis may be regarded as a pathogenic risk factor for cerebral and intestinal diseases.

The gut microbiome and its metabolites, such as short-chain fatty acids (SCFAs) and neurotransmitters, can change the CNS microenvironment, adjust immune responses, and disturb the endocrine system (Dalile et al., 2019). Epidemiological studies have further demonstrated that dysbiosis in microglial activation and amino acid metabolism pathways could be triggered by an unbalanced intestinal flora and result in extensive tissue remodeling and disruption of the antitumoural immune response in the brain tumor microenvironment. In addition, one animal study further revealed that alterations in microbial composition could promote glioma pathophysiology in rats (Patrizz et al., 2020). Although increasing evidence suggests a close connection between brain tumors and abnormalities in gut microbial function, evidence from only animal or epidemiological studies is insufficient to identify the exact correlations and mechanisms in humans. Hence, understanding of the microbial composition and the composition of metabolites in the gut microbiota in brain tumor patients is still unclear. Therefore, an intense interest in characterizing the functional intestinal flora in brain tumor patients has arisen.

In this study, we applied 16S rRNA gene sequencing to fecal samples from healthy controls and brain tumor patients to characterize the intestinal microbial community and explore potential microbial biomarkers for early diagnosis and potential targeted therapy.

## Methods

### Research Design and Participants

In this research, a total of 158 participants were recruited by the Department of Neurosurgery, Clinical Medical College of Yangzhou University (Jiangsu, China) from June 2020, to August 2021, including 101 patients with brain tumors and 57 healthy controls. All participants received conventional medical and stool examinations. Their clinical data were recorded, including age, sex, body mass index (BMI), etc. All the patients and healthy controls were Jiangsu Han Chinese, with similar living areas and eating habits. The diagnoses of all patients were confirmed by postoperative pathology. In the brain tumor group, 23 patients were diagnosed with gliomas, 32 with meningiomas, 24 with pituitary tumors, 13 with brain metastases, and 9 with other brain tumors based on postoperative histopathology. All the patients were newly diagnosed with brain tumors and at the early stage of disease.

Exclusion criteria for all subjects were as follows: age of less than 18 years or older than 85 years; advanced stage disease; family genetic history of brain tumors, inflammatory bowel disease (IBD), ulcerative colitis (UC) and other serious gastrointestinal diseases; history of treatments, such as surgery, chemoradiotherapy and pharmacological therapy, before sampling; presence of other coexisting benign or malignant tumors; and abnormal intestinal habits or severe gastrointestinal diseases; history of receiving antibiotics, probiotics or prebiotics within two months before sampling. Our research was approved by the Ethics Committee of the Clinical Medical College of Yangzhou University (2021ky-007-1), and the present study was registered at the Chinese Clinical Trial Register (ChiCTR2100044256). Written informed consent was obtained from the brain tumor patients and the healthy controls.

### Fecal Sample Collection and DNA Extraction For Microbiome Analysis

All fecal specimens were collected by sterile cotton swabs within 2 hours of hospital admission, immediately placed in a proprietary preservation solution and then immediately stored at -80°C until further testing. Genomic DNA was extracted with a PowerMax extraction kit and stored at -80°C. The quantity and quality of DNA were determined by a NanoDrop ND-1000 spectrophotometer (Thermo Fisher Scientific, Waltham, MA, USA).

### 16S rRNA Amplicon Pyrosequencing

PCR amplification of the bacterial 16S rRNA gene V4 region was performed using the forward primer 515F (5’-GTGCCAGCMGCCGCGGTAA-3’) and the reverse primer 806R (5’-GGACTACHVGGGTWTCTAAT-3’). Sample-specific paired-end 6-bp barcodes were incorporated into the TruSeq adaptors for multiplex sequencing. Thermal cycling consisted of initial denaturation at 98°C for 30 s, followed by 25 cycles of denaturation at 98°C for 15 s, annealing at 58°C for 15 s, and extension at 72°C for 15 s, with a final extension at 72°C for 1 min. PCR amplicons were purified by AMPure XP Beads (Beckman Coulter, Indianapolis, IN) and quantified using a PicoGreen dsDNA Assay Kit (Invitrogen, Carlsbad, CA, USA). After the individual quantification step, amplicons were pooled in equal amounts, and paired-end 2×150 bp sequencing was performed using the Illumina NovaSeq6000 platform.

### Sequencing Data Analysis

The paired-end reads were assigned to respective samples using specific barcodes, which were cut off with primer sequences together. Then, the truncated reads were merged using the FLASH (Fast Length Adjustment of SHort reads) tool. The Quantitative Insights Into Microbial Ecology (QIIME, v1.9.0) pipeline was employed to filter the raw tags, and then high-quality clean tags were obtained. Chimeric sequences were filtered using Vsearch (Version 2.4.4) software. Sequences with similarity thresholds greater than 97% were allocated to one operational taxonomic unit (OTU) by using CD-HIT software online (v4.6.1). Classification of representative sequences for each OTU was performed, and the taxonomic data were assigned to each sequence by the Ribosomal Database Project (RDP) classifier 2.10.1. To determine the phylogenetic differences in dominant OTUs, multiple sequence alignments were carried out using Python Nearest Alignment Space Termination (PyNAST) software. Sequences were aligned for phylogenetic analysis after the taxonomic assignment of OTUs. OTU-level alpha diversity indices (Shannon, Simpson, Chao1) were calculated to evaluate the complexity of microbial diversity in each sample using the OTU table in QIIME. Beta diversity analysis was performed to investigate the structural variation in microbial communities across samples using UniFrac distance metrics, and the data were visualized *via* principal coordinate analysis (PCoA) and nonmetric multidimensional scaling (NMDS). The analysis of similarities (ANOSIM) was calculated to evaluate the statistical significance. The influence of differentially abundant taxa was evaluated using linear discriminant analysis (LDA) coupled with the effect size (LEfSe) method. KEGG (Kyoto Encyclopedia of Genes and Genomes) and the Phylogenetic Investigation of Communities by Reconstruction of Unobserved States (PICRUSt) database were used to conduct pathway enrichment analysis. The output file was further analyzed using Statistical Analysis of Metagenomic Profiles (STAMP, v2.1.3) software. BugBase is a tool for measuring high-level phenotypes in the microbiome. (https://bugbase.in/).

### Statistical Analyses

Clinical and sequencing data analyses were mainly performed using SPSS (v19.0) and R packages (v3.2.0). The continuous variable data are displayed as the mean ± standard deviation, and an independent t test was conducted to calculate the differences between groups. Moreover, we further compared microbial abundance and diversity in fecal samples between brain tumor patients and healthy controls using the Wilcoxon rank-sum test. The differences in the UniFrac distances for pairwise comparisons between groups were determined using Student’s t test or the Monte Carlo permutation test. The genus-level heatmaps were also drawn on the basis of the nonparametric Wilcoxon test (q< 0.1). In addition, the chi-square test or Fisher’s exact test was also used for categorical variables. Finally, *p* values adjusted using an FDR (false discovery rate) of less than 0.05 were considered statistically significant in the comparisons of groups.

## Results

### Summary of Clinical Parameters

In the current study, we enrolled 101 patients with brain tumors (T group), as well as 57 matched healthy controls (C group). The clinical variables of the two groups were completely matched (**Table 1**), indicating that no established confounding factors affected group discrimination prior to the design of this experiment. Moreover, to determine whether there were differences in the gut microbiota associated with different brain tumors, we divided the brain tumors into benign brain tumors (BBTs, mainly meningiomas and pituitary tumors, 65 cases) and malignant brain tumors (MBTs, gliomas and metastatic brain tumors, 36 cases) and included the 57 matched healthy controls (HCs) to conduct the subgroup analysis.

**Table 1:** Descriptive data of included subjects in the study.

Characteristics	Brain tumours (T)	Benign brain tumours (BBT)	malignant brain tumours (MBT)	Healthy Controls (C)	BBT vs. MBT	T vs. C
	(n = 101)	(n = 65)	(n = 36)	(n = 57)	*p*	*p*
**Age (Mean ± SD)**	57.34±10.68	55.92±10.50	59.89±9.80	57.05±11.46	0.462	0.765
**Gender**					0.322	0.925
Female	57	34	23	33		
Male	44	31	13	24		
**BMI**	23.91±3.24	24.12±3.17	23.53±3.38	23.71 ± 3.47	0.462	0.628
**Smoking**					0.145	0.417
Absence	72	46	26	44		
Presence	29	19	10	13		
**Drinking**					0.786	0.505
Never	59	40	19	36		
<1 standard drink per day	29	19	10	17		
≥1 standard drink per day	13	6	7	4		
**Hypertension**					0.765	0.485
Negative	78	55	23	47		
Positive	23	10	13	10		
**Diabetes**	69	45	24	46	0.421	0.254
**Hypercholesterolemia**	21	14	7	5	0.553	0.254
**Excrement regularity**					0.754	0.626
Yes	82	58	24	47		
No	19	7	12	10		
**Tumor location**					0.024	
Left	48	38	10	–		
Right	45	27	18	–		
Diffuse	8	0	8	–		
**Tumor maximum diameter (Mean ± SD)**	55.3±15.2	50.5±14.3	61.2±16.4	–	<0.01	

### Diversity of the Gut Microbiota of Brain Tumor Patients

**Figure 1:**
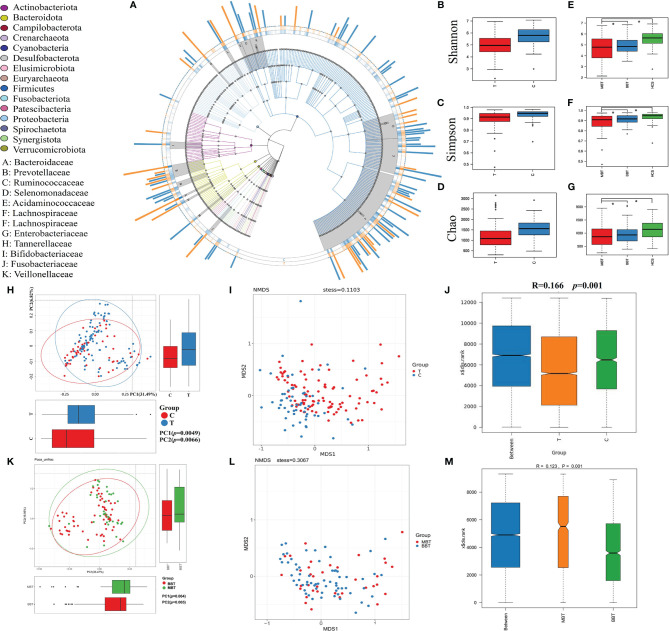
The general distribution and microbial diversity in the gut microbiota of all participants. Distribution and species tree of the intestinal bacterial community **(A)**. The differences in alpha diversity were estimated by the Shannon, Simpson, and Chao1 indices. Shannon indices between the two groups (brain tumor group and healthy group, **B**) and three groups (benign brain tumor group, malignant brain tumor group and healthy group, **E**). The Simpson indices of the two groups **(C)** and three groups **(F)**. The Chao1 indices of the two groups **(D)** and three groups **(G)**. The variation in these data could be analyzed by unpaired t tests or Wilcoxon rank-sum tests. The asterisk suggests *p <*0.05. Principal coordinate analysis (PCoA) based on the unweighted UniFrac distances and nonmetric multidimensional scaling of bacterial beta diversity between the brain tumor group and the healthy group **(H, I)** and between the MBT group and BBT group **(K, L)**. Each point represents a sample. Analysis of similarities (ANOSIM) between the brain tumor group and the healthy group **(J)** and between the MBT group and the BBT group **(M)**. We defined p=0 when p<0.000001.

The gut microbiota was evaluated using 16S rRNA sequencing. A total of 19,948,889 high-quality 16S rRNA reads were identified, with a median read count of 130,521 (ranging from 95356 to 138015) per sample. After taxonomic assignment, 5469 operational taxonomic units (OTUs) were obtained, including 12 phyla, 59 families and 147 genera of gut microbes (**Figure 1A**). Alpha diversity indices, which were analyzed using sampling-based OTUs, were further calculated to evaluate the differences in the microbial diversity between each group. The results indicated that the gut microbial alpha diversity of brain tumor patients was significantly lower than that in healthy subjects in terms of the Shannon, Simpson and Chao1 indices (**Figures 1B–D**; *p*=0, 0.0001, 0). The subgroup analysis showed no difference in alpha diversity between the BBT and MBT groups (**Figures 1E–G**; *p=*0.32, 0.41, 0.64). The beta diversity was evaluated to illustrate the differences in the structural variation in microbial communities between groups. Principal coordinate analysis (PCoA) based on unweighted UniFrac distance showed that the gut microbiota of brain tumor patients clustered somewhat separately from that of healthy subjects (**Figure 1H**, PC1 = 0.0049, PC2 = 0.0066). Nonmetric multidimensional scaling (NMDS) analysis based on Bray–Curtis distances between groups revealed a significant deviation in microbial genera (STRESS=0.1103<0.2, **Figure 1I**). The subgroup analysis showed no obvious difference in beta diversity between the MBT group and the BBT group. (PC1 = 0.064, PC2 = 0.065, **Figure 1K**; STRESS=0.3067>0.2, **Figure 1L**). The results of similarities analysis (ANOSIM) revealed that each group has good representativeness with small intragroup differences, so the grouping is meaningful between the brain tumor group and the healthy group (ANOSIM, r=0.166>0, p= 0.001, unweighted UniFrac, **Figure 1J**), and the same results were obtained between the MBT and BBT groups (ANOSIM, r=0.123>0, p= 0.001, unweighted UniFrac, **Figure 1M**).

### Alterations in the Composition of the Fecal Microbiota in Brain Tumor Patients

To investigate the intestinal microbial community features of brain tumor patients, the relative taxon abundance of the microbiota was compared among different groups, and considerable variability was observed.

**Figure 2:**
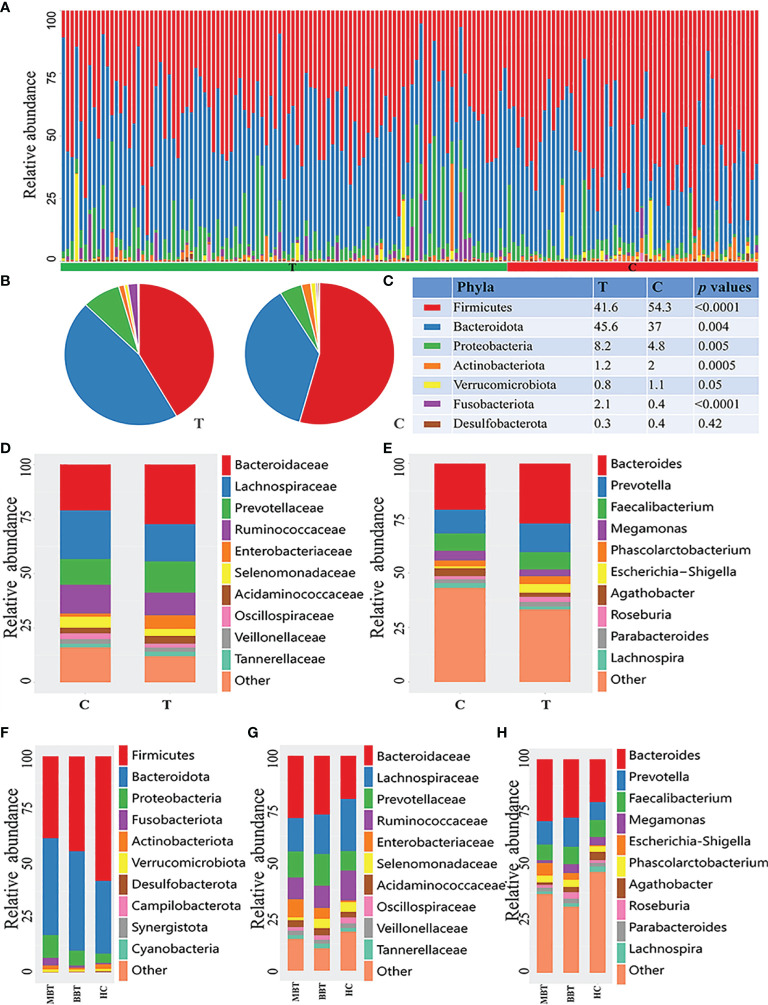
Composition of microbial community at different levels of different groups. The phylum-level distribution of all samples **(A),** brain tumor group and healthy group **(B)**. The proportion and comparation of the top seven bacteria at phylum level of T and C groups **(C)**. Each color represents a kind of bacteria. The microbial family **(D)** and genera **(E)** between T and C groups. And the analysis of subgroups at the level of phylum **(F)**, family **(G)** and genus **(H)** in malignant brain tumours (MBT), benign brain tumours (BBT) and healthy group (HC).

At the phylum level, the brain tumor group had a higher abundances of *Bacteroidetes* (45.6% vs. 36.9%, *p*=0.004), *Fusobacteria* (2.13% vs. 0.37%, *p*<0.001) and *Proteobacteria* (8.23% vs. 4.77%, *p=*0.005) and lower relative abundances of *Firmicutes* (41.58% vs. 54.31%, *p<*0.0001) and *Actinobacteria* (1.22% vs. 1.96%, *p=*0.0005) than those of the healthy controls. Hence, the *Firmicutes/Bacteroidetes* (F/B) ratio was decreased significantly in the tumor group (*p*<0.001, **Figures 2A–C**). Moreover, *Fusobacteria* and *Proteobacteria* showed a higher abundance in the MBT group than in the BBT group (**Figure 2F**).

At the family level, the brain tumor-enriched species included *Bacteroidaceae* (p=0.027), *Fusobacteriaceae* (*p*<0.001) and *Enterobacteriaceae* (*p*<0.001), whereas *Bifidobacteriaceae* (*p*<0.001), *Lachnospiraceae* (p=0.002), and *Akkermansiaceae* (*p*=0.042) were more prevalent in the healthy control group (**Figure 2D** and **Supplementary Table 1**). Moreover, there was an obviously higher abundance of *Enterobacteriaceae* and a lower abundance of *Lachnospiraceae* in the MBT group than in the BBT group (**Figure 2G**).

The genus-level characterization results were more complicated, and the relative abundances of the top 60 dominant taxa in all samples were further calculated by clustering analysis and displayed in the heatmap shown in **Supplementary Figure 1**. After independent t test and Wilcoxon rank-sum test, a total of 31 genera were identified as significantly differentially abundant between each group. Of these discriminatory taxa, *Bacteroides* (*p*=0.027), *Escherichia/Shigella* (*p*<0.001), *Fusobacterium* (*p*<0.001), *Sutterella* (*p*=0.047), and *Ruminococcus gnavus group* (*p*<0.001) showed visibly higher abundances in the tumor patient group than in the healthy control group (**Figure 2E** and **Supplementary Table 2**). Moreover, the abundances of the genera *Roseburia* and *Megamonas* were lower, and the abundance of *Escherichia/Shigella* was higher in the MBT group than in the BBT group (**Figure 2H**).

### Potential Microbial Biomarkers for Patients With Brain Tumors and Healthy Controls

**Figure 3:**
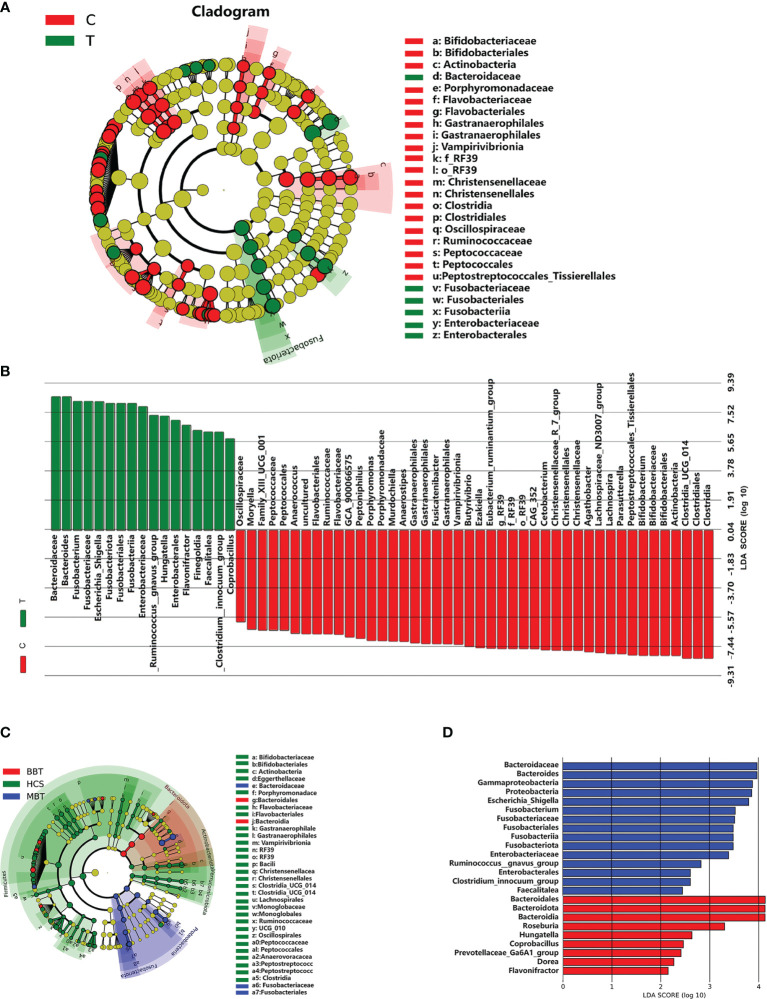
Differential enriched bacterial taxa between different groups. Linear discriminant analysis (LDA) integrated with effect size (LEfSe) was used to identify the taxa with the greatest differences in abundance between different groups. Cladogram indicating the phylogenetic distribution of microbiota correlated with the T or C groups **(A)**. Histogram of the LDA scores computed for differentially abundant taxa between T and C groups **(B)**. Meanwhile, the enriched taxa in the malignant brain tumours, benign brain tumours and the healthy were also displayed in the cladogram **(C)**, and the differences in abundance between the malignant and benign brain tumours **(D)**. The central point represents the root of the tree (microorganism), and each ring represents the next lower taxonomic level. The diameter of each circle represents the relative abundance of the taxon, only the taxa satisfied an obvious LDA threshold value of >2 are presented.

Linear discriminant analysis (LDA) effect size (LEfSe) analysis was performed to generate a cladogram to identify the specific microbiota of the brain tumor patient group and the healthy control group (**Figure 3A**). A variety of opportunistic pathogens, including *Fusobacterium* (*Fusobacteriaceae, Fusobacteria*), *Enterobacteriaceae* (*Enterobacterales*) and *Escherichia/Shigella*, were all significantly overrepresented in brain tumor patients (*p*<0.01, Wilcoxon rank-sum test; LDA, log 10>5), whereas *Parasutterella*, *Bifidobacterium* (*Bifidobacteriales*, *Actinobacteria*) and *Lachnospira* were significantly enriched (LDA, log 10>7) in the healthy controls (**Figure 3B**). In addition, the unique bacterial biomarkers for different groups (MBT, BBT, HC) are illustrated in **Figures 3C, D**. We found abundant common biomarkers for healthy subjects, while the genera *Roseburia* and *Hungatella* were enriched in the BBT patients, and *Escherichia/Shigella* and *Fusobacterium* were the most abundant in the MBT patients.

### The Gut Flora-Based Signature Discriminates Tumor Patients From Healthy Subjects

**Figure 4:**
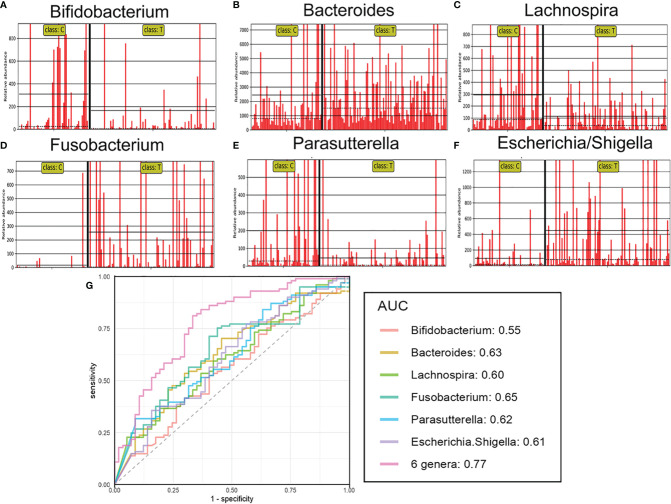
The relative abundance and predictive ability for brain tumours of differential genera. The relative abundance of the differential genera such as Bifidobacterium **(A)**, Bacteroides **(B)**, Lachnospira **(C)**, Fusobacterium **(D)**, Parasutterella **(E)**, Escherichia/Shigella **(F)** in each sample. Receiver operating characteristic (ROC) curves for the differential genera alone or in combination **(G)**, which was calculated to discriminate brain tumor patients from healthy controls. AUC: the area under the receiver operating characteristic curve.

Several differentially abundant taxa of the gut flora were revealed in the brain tumor group, and then we assessed the possible value of using six abundant genera, including *Bifidobacterium, Bacteroides, Lachnospira, Fusobacterium, Parasutterella*, and *Escherichia/Shigella*, as biomarkers. The differential features of these genera showed obvious intergroup changes (**Figures 4A–F**). Then, each of six differentially abundant bacteria was used to generate receiver operating characteristic (ROC) curves to predict classification of the groups, and an area under the curve (AUC) ranging from 0.55 to 0.65 was obtained (**Figure 4G**). In addition, we discovered that combining all six genera significantly improved the predictive performance (AUC: 0.77). Hence, this microbial biomarker panel could be used to discriminate brain tumor patients from healthy controls.

### The Functional Profile of the Intestinal Flora in Patients With Brain Tumors Was Changed

**Figure 5:**
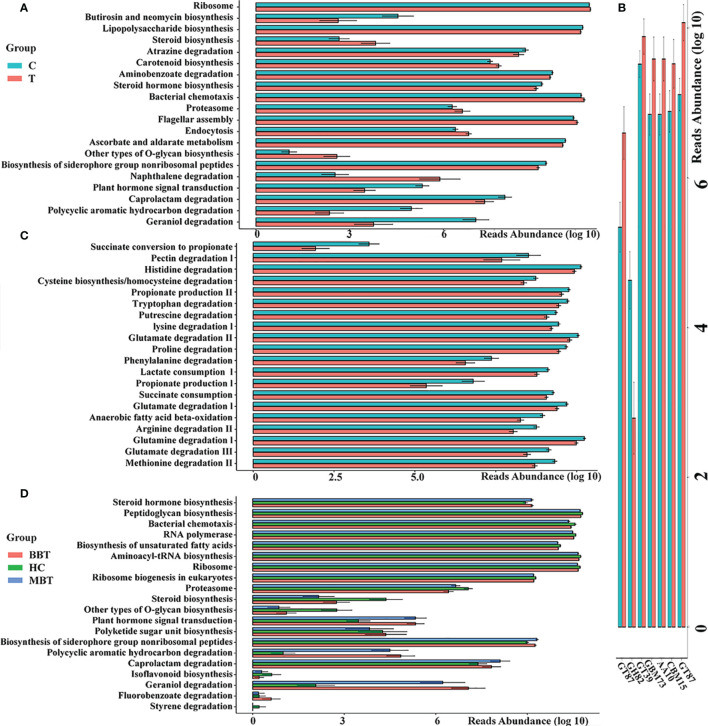
Predicted metagenome function of gut microbiota between different groups. PICRUSt built-in database and Carbohydrate-Active enZYmes (CAZy) Database were referenced to evaluate different bacterial functions in the brain tumours and the healthy groups. Comparisons between the groups for the most twenty significantly different KEGG pathways **(A)** and seven Carbohydrate-active enzymes (CAZy, **B**) are displayed. The gut metabolic modules of KEGG pathways **(C)**. The subgroup analysis of KEGG pathways **(D)**. All the differences in this graph show a *p* value less than 0.05.

An imbalanced microbiota could induce systematic metabolic dysfunction, whereas metabolic dysregulation could in turn alter intestinal flora composition (Song et al., 2020; Fan and Pedersen, 2021). To investigate the metabolic and functional alterations in gut microbial communities, each OTU was aligned into the PICRUSt built-in reference database and the Carbohydrate-Active enZYmes (CAZy) database. The analysis based on the PICRUSt and CAZy databases identified the top 20 KEGG pathways and top 7 CAZys with significantly differential abundance between groups. As presented in **Figures 5A, B**, the pathways of bacterial motility, membrane transport, biosynthesis of essential substances and energy metabolism were less abundant, while the pathways involved in the accumulation of toxic substances were more abundant in the tumor group than in the control group. According to the results of the gene annotation performed based on the KEGG Orthology database, the clustering of gut metabolic modules mainly involved the degradation of neurotransmitters, such as amino acids or their precursors, and the brain tumor group showed a significantly higher abundance than that of the healthy control group (**Figure 5C**). Subgroup analysis showed that the MBT group had a higher degree of change than that in the BBT group in many basic KEGG pathways (**Figure 5D**).

### The Associations Between the Various Bacterial Communities Were Complicated

**Figure 6:**
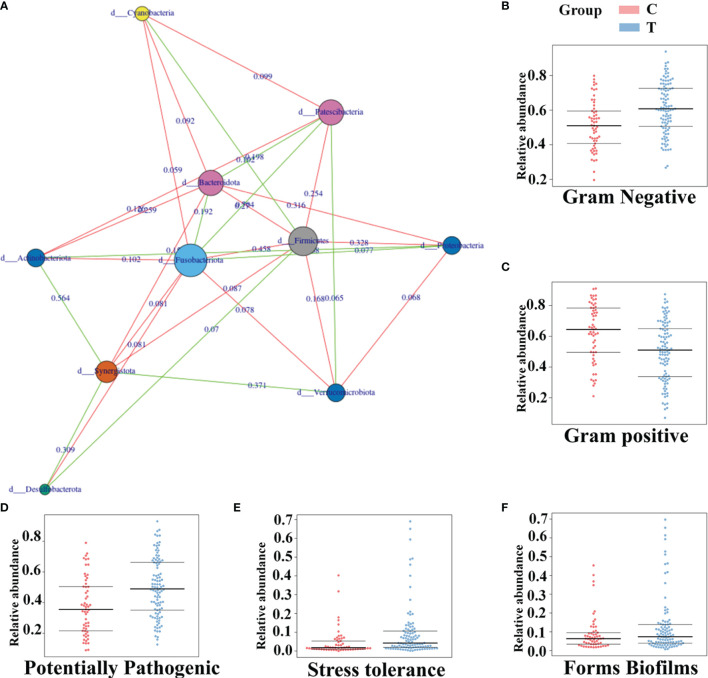
The phenotypic characteristics of gut microbiota and the connection among them in brain tumor patients. The connection among different gut microbiota at the phylum level of brain tumor patients **(A)** Green represents positive correlation and red shows the negative. And the value on the line represents the association strength. The software of Bugbase was used to predict the phenotypic characteristics of the intestinal flora about Gram Negative **(B)** Gram positive **(C)** Potentially Pathogenic **(D)** Stress tolerance **(E)** Forms Biofilms **(F)** Each spot is a sample.

In the tumor group, there were conspicuous dynamic relationships among the 10 phyla, including 11 positive relationships and 18 negative relationships. The abundances of *Firmicutes* were all strongly negatively correlated with the abundances of *Bacteroidetes, Proteobacteria* and *Fusobacteria*. The abundance of *Fusobacteria* was positively correlated with the abundances of *Bacteroidetes* and *Proteobacteria* but negatively correlated with the abundances of *Verrucomicrobia* and *Actinobacteria* (**Figure 6A**). To explore the underlying reasons for these relationships, Bugbase software was used to identify the phenotypic characteristics of the microflora, and the results were as follows in the comparison of the brain tumor group and the healthy group: the number of gram-positive bacteria was evidently lower (*p*=0.00025), and the number of gram-negative bacteria (*p*<0.001) and opportunistic pathogens (*p*=0.00011) and the degree of oxidative stress tolerance (*p*=0.00012) showed the opposite trend, while there was no significant difference in biofilm formation (*p*=0.19). (**Figures 6 B–F**);

## Discussion

The gut harbors a broadly diverse microbiota that can produce an extremely wide range of small molecules that affect several vital pathways associated with immune balance and the homeostasis of neurological function (Cryan et al., 2019; Song et al., 2020). In recent years, accumulating evidence has revealed that the gut microbiota and its metabolites contribute to neurological disorders and tumorigenesis in various systems (Wong and Yu, 2019; Di Modica et al., 2021). The characteristics and regulation of the gut microbiota have gradually become emerging tools to diagnose and treat tumor patients.

Recent studies have suggested that a strong relationship between the microbiota and brain tumors may exist. Yuqi Wen et al. proved that the oral microbiota could be a useful biomarker to distinguish glioma patients from healthy individuals (Cryan et al., 2020). A study from Yingying Lyu et al. (Patrizz et al., 2020) also revealed that the gut microbiota could regulate the immune environment of gliomas by the gut-brain axis. However, most related studies have a small sample size. In our present study, we recruited 101 patients with brain tumors (which could be divided into BBT and MBT groups) and 57 matched normal controls to explore the underlying relationships between the gut microbiome and brain tumors. The results of our comparison of the brain tumor and healthy groups suggested that the brain tumor group presented a microbial ecosystem with low density and loss of microbial diversity. The alpha and beta diversity analyses showed that the microbial structure of brain tumor patients was also obviously different from that in the healthy group. Moreover, we investigated the differences between the BBT and MBT groups. However, the results indicated similar alpha and beta diversity between the BBT and MBT groups. In addition to microbial diversity, we focused on microbial gene functions and on the development of a potential biomarker panel that could be used to distinguish brain tumor patients from healthy controls and aimed to identify significant differences in the gut microbiota of BBT and MBT patients. Hence, this study may provide evidence of substantial changes in the gut flora of benign and malignant brain tumor patients.

Significant changes in brain tumor-associated gut microbiota compositions were observed in our research. The gut microbial composition may be obviously relevant to brain tumor malignancy. For example, at the phylum level, the intestinal microbiota showed the lowest abundance of *Firmicutes* in the MBT group and the lowest abundance of *Actinobacteria* in the BBT group, unlike those in the healthy group. Several studies have shown that *Firmicutes* can regulate anti-inflammatory activities and apoptosis by producing butyrate (Dalile et al., 2019; Tran and Mohajeri, 2021; Qu et al., 2021). *Actinobacteria* are valeric acid-associated microbes and have also been demonstrated to be depleted in colorectal tumors (Yang et al., 2019). In the MBT group, we revealed increased abundances of *Fusobacteria* and *Proteobacteria*. *Fusobacteria*, which are opportunistic pathogens, have been reported to promote tumor cell proliferation by regulating T regulatory cells and the activation of autophagy (Chen et al., 2020). In addition, most genera of *Proteobacteria* have been classified as gram-negative pathogens (Vaz-Moreira et al., 2017; Guan et al., 2018). The abnormal increase in *Proteobacteria* abundance indicates that the intestinal epithelial barrier is broken and more susceptible to infection (Yoo et al., 2020). In the BBT group, we found that *Bacteroidetes* was especially enriched. *Bacteroidetes* has been repeatedly reported to be essential for the host by performing metabolic conversion. The imbalanced *Bacteroidetes* abundance could induce neuroinflammation by the production of excessive propionate (Li et al., 2019). This chemical substance has a dose-regulated influence on brain cell neurotoxicity and microglial activation (El-Ansary et al., 2012). Moreover, the excessive abundance of *Bacteroidetes* bacteria in brain tumors reduced the ratio of *Firmicutes* to *Bacteroidetes*. A lower F/B ratio is associated with the dysbiosis of host systemic inflammation and immunity by decreasing the concentration of circulating SCFAs (Dalile et al., 2019; Liu et al., 2019; Liu et al., 2019). Hence, a generally imbalanced gut microbial ecosystem associated with the degree of malignancy was revealed in brain tumor patients.

Various additional pathogenic bacteria were demonstrated to be enriched at the family and genus levels, indicating that the characteristics are more complicated and significantly varied in patients with brain tumors. For example, the abundance of the genus *Sutterella* was increased in the brain tumor group. A study suggested that *Sutterella* could drastically reduce the diversity of the microbial ecosystem by degrading IgA and promoting tumor progression (Kaakoush, 2020). Except for that of *Sutterella*, the abundances of almost every genus revealed a great increase in brain tumor patients, and this microbial signature was then proven to be a tumor biomarker with different tumor-promoting effects. Contrary to the results of previous studies that identified *Bacteroides* as a probiotic for enhancing anti-CTLA4 immune checkpoint efficacy (Wexler, 2007; Liu et al., 2019), excessive *Bacteroides* abundance could be neurotoxic (Wexler, 2007; Cattaneo et al., 2017). In addition, it has been reported that *Bacteroides* and *Clostridia* could possess complicated pathways to metabolize tryptophan and modulate this metabolite by regulating TPH1 (a key rate-limiting enzyme) (Yano et al., 2015; Chen et al., 2021; Taverniti et al., 2021). The abundance of serotonin could have a bidirectional effect on brain tumor growth (Dehhaghi et al., 2020). The genera *Escherichia/Shigella* and *Fusobacterium* were also revealed as biomarkers for malignant brain tumors. Both of these genera have been proven to be associated with cancers. They may promote MBT proliferation by increasing the levels of neurological inflammation and regulating the expression and activity of DNA methyltransferase, respectively (Xia et al., 2020). In the BBT group, the genus *Roseburia*, which has potential for use as a probiotic for the treatment of diseases, was significantly enriched. Its ability to modulate the brain functional connectivity pattern by tumor necrosis factor-alpha (TNF-α) may be the reason for this observation. Moreover, many probiotic bacteria were also revealed as biomarkers for the healthy group. The genera *Bifidobacterium* and *Parasutterella* are the core components of the gut microbiota, and the loss of these genera could contribute to the imbalance of the immunity, neurohormone and metabolic systems (Kang et al., 2017; Jena et al., 2018). Moreover, both the *Clostridia* and *Lachnospira* genera could inhibit tumor proliferation by producing butyric acid (Calvo-Barreiro et al., 2021). All the evidence suggests that MBT patients possess more abundant pathogens and that specific constituents of the gut microbiome may be applied to diagnosis or used as therapeutic targets and even for fecal microbiota transplantation. Furthermore, the alteration of the gut bacterial population associated with brain pathology, which can be assessed using metrics such as alpha and beta diversity, may provide potential predictive information for tumor progression and recurrence.

In addition, we should pay attention not only to the specific constituents of the gut microbiome in brain tumor patients but also to the interactions among them. We speculate that the underlying dynamic connections among the microbiome may play an essential role in tumor growth. For example, the lipopolysaccharide of gram-negative bacteria (mostly from *Proteobacteria*) could promote the proliferation of facultative anaerobic pathogens by providing them with energy (Maldonado et al., 2016; Yoo et al., 2020). This relationship suggests a disruption in the oxygen levels in the intestinal environment, which may explain the increased abundance of *Enterobacteriaceae* and the reduction in the abundance of gut strict obligate anaerobes, including *Firmicutes* and *Akkermansiaceae* (Yoo et al., 2020). Then, the increased *Enterobacteriaceae* abundance could induce neutrophil transepithelial migration and the depletion of SCFA-producing bacteria (e.g., *Bifidobacteriaceae* and *Clostridia* genera). Moreover, the depletion of SCFA-producing bacteria may inhibit their ability to limit the colonization of *Enterobacteriaceae via* intestinal pH reduction (Behnsen, 2017; Yoo et al., 2020), which is shown in **Figure 6**.

It is known that an imbalanced microbial structure can induce dramatic metabolic disorders. For example, the levels of SCFAs generated by various bacteria, such as *Bifidobacterium* and *Clostridium*, may be decreased in MBT and BBT patients. By upregulating the levels of tight junction proteins, SCFAs can regulate the permeability of the gut tract and promote the release of many cytokines and molecules in the blood circulation and CNS (Feng et al., 2018). SCFAs participate in the process of epigenetic modulation by inhibiting histone deacetylase, which promotes the development of MBTs (Tran and Mohajeri, 2021). For immune profiling, SCFAs can activate G-protein-coupled receptors (GPCRs), such as GPR43 and GPR109A, which have a close relationship with microglial cell morphology and growth hormone secretion in pituitary cells (Fernandes et al., 2020; Silva et al., 2020). In addition, SCFAs modify the metabolism and activity of brain tumor-associated immune cells (Bachem et al., 2019). Therefore, dynamic dysbiosis of all the constituents of the gut microbiome, rather than of specific pathogens, could promote tumor development in both MBT and BBT patients.

The analysis of KEGG pathways suggested that the disturbance in the intestinal microflora in brain tumor patients is closely linked with the dysbiosis of several basic physiological processes, mainly including metabolism, cellular processes, and environmental information processing. These data also demonstrated that the synthesis and decomposition of carbohydrates have less abundant roles in the gut microbiota of brain tumor patients. The dysregulation of energy metabolism has been reported to be essential for the pathogenesis of tumors. In summary, these abnormal changes further suggest potential metabolic and immune alterations in brain tumor patients. For example, carotenoids with the ability to activate intracellular signaling cascades could promote inflammation or oxidative stress responses by influencing gene expression and protein translation, and the levels of these compounds were found to be increased in tumor patients (Reboul, 2019). In addition, the inhibition of the bacterial chemotaxis and endocytosis pathways suggests the weakening of bacterial motility and membrane transport, which is consistent with the feature of low gut microbial diversity in brain tumor patients. Moreover, the excessive degradation of amino acids such as arginine and glutamate in the tumor group could result in an increase in neurotransmitter levels (Tran and Mohajeri, 2021) and then activate the uncontrolled proliferation and diffusion of tumor cells (Jiang et al., 2020). Glutamate is an important source of ⍺-KG, which participates in metabolic process that occurs after IDH1/2 mutation and is associated with the methylation of DNA (Lyu et al., 2021). A study indicated that glutamine could be derived from glutamate and promote tumor autophagy, resulting in increased tumor energy metabolism and eventually contributing to the proliferation of brain tumors (Márquez et al., 2017). In addition, neuroactive metabolites, such as nitric oxide and polyamines, can be derived from arginine, and they can be translocated into the brain and lead to the metastasis and proliferation of brain tumors by modulating spermidine/spermine acetyltransferase or ornithine decarboxylase (Dehhaghi et al., 2020). Moreover, in the subgroup analysis, we found that the MBT group showed a higher degree of change than that in the BBT group, especially in genetic information processing pathways, such as aminoacyl-tRNA biosynthesis, which suggested that the epigenetic environment is most damaged in the MBT patients.

In summary, the interactions between the gut microbiota and brain tumors are complicated. Unfortunately, current understanding of these interactions is still insufficient. However, all the evidence suggests that brain tumors are fundamentally metabolic diseases and often present with coexisting disorders of the gut microbiota (Liu et al., 2019). Therefore, the perturbations in the microbiome–metabolome interface can be further understood, and several underlying diagnostic or therapeutic targets may be identified in brain tumor patients. Furthermore, it is not definite that the gut microbiota performs its function only by any one of these interactions, and the brain tumor environment and the formation of subtypes may be influenced by alterations in these relationships. We speculate that the process is caused by the tumor-promoting metabolic products and toxins regulating immune and inflammatory responses in the host.

Finally, it has been reported that medicines can affect the progression of tumors by regulating the microbial community (D'Alessandro et al., 2020); moreover, changes in the bacterial community can also improve the efficacy of antitumour drugs and reduce their side effects (Vivarelli et al., 2019), which may provide potential strategies for the treatment of brain tumors. Probiotics or specific synthetic metabolites could be prescribed to combat dysbiosis in tumor patients, especially MBT patients receiving traditional treatments. The relationships between the gut microbiome and brain tumors in this study could provide new inspiration for the investigation of the potential function of the microbiota in the diagnosis and treatment of brain tumors. Hence, larger and more rigorous trials should be designed to explore this essential point.

There were some limitations in our study. First, the nature of the case–control study makes it difficult to dissect the mechanisms and establish a longitudinal view of the relevance of the findings, which is essential to confirm the dynamics of the microbiota and to assess the changes in the intestinal flora of brain tumor patients. Second, no accurate metabolic data were obtained to provide a complete picture of how gut microbiota and corresponding metabolic processes might be impacted during the disease state. Moreover, the subgroup analysis and the associations of brain tumor patients with disease staging were not performed in detail. In addition, to determine the discriminatory power of gut microbial biomarkers, separate test and validation cohorts are required in subsequent studies. The sequencing method has inevitable inherent limitations, such as amplification bias. Finally, it is known that the dysregulation of intestinal bacteria is associated with brain tumors, but we cannot exclude the possibility that microbiome changes may be a passive bypby-product of tumor progression. Future research could include corresponding experiments to investigate immune profiling and microbiota-based biomarkers during different tumor stages, establishing a more accurate prediction model for diagnosis and monitoring the stage of diseases.

## Conclusions

Gut microbial dysfunction was demonstrated to yield a group of microbial markers for brain tumor patients. The dysbiosis of several pathogens, including *Bifidobacterium, Bacteroides, Lachnospira, Fusobacterium, Parasutterella*, and *Escherichia/Shigella*, may be an underlying risk for brain tumor development. The use of gut microbiota-based biomarkers could be regarded as a promising noninvasive means to detect brain tumors. However, a characteristic xenograft based on the target microbiome should be established and then incorporated into *in vivo* and *in vitro* studies, which is crucial to elucidate the mechanisms of intestinal flora dysbiosis in brain tumor growth.

## Data Availability Statement

The datasets presented in this study can be found in online repositories. The names of the repository/repositories and accession number(s) can be found below: NCBI BioProject - PRJNA807001.

## Ethics Statement

The studies involving human participants were reviewed and approved by the Ethics Committee of the Clinical Medical College of Yangzhou University. The patients/participants provided their written informed consent to participate in this study.

## Author Contributions

GL, YL and HZ conceptualized, designed, and supervised the project. YL, HJ, and XW conducted the data analysis and wrote the manuscript. HJ, ZW, and QM conducted the experiments. XW, LD, XL, YQ and YH collected the samples. All authors read and approved the manuscript.

## Funding

This study was supported by grants from the National Natural Science Foundation of China (No. 82172603) and the Natural Science Foundation of Jiangsu Province (No. BK20190241)

## Conflict of Interest

The authors declare that the research was conducted in the absence of any commercial or financial relationships that could be construed as a potential conflict of interest.

## Publisher’s Note

All claims expressed in this article are solely those of the authors and do not necessarily represent those of their affiliated organizations, or those of the publisher, the editors and the reviewers. Any product that may be evaluated in this article, or claim that may be made by its manufacturer, is not guaranteed or endorsed by the publisher.

## Supplementary Material

Supplementary Figure 1 Heat map of the relative abundances of the top 60 dominant taxa at genus level in all samples. The genera were displayed from higher abundance (in red) to lower abundance (in blue).
