# Supplementary figures and images for: Crosstalk Between the Gut and Brain: Importance of the Fecal Microbiota in Patient With Brain Tumors
Source: Front Cell Infect Microbiol. 2022 Jun 17;12:881071. doi: 10.3389/fcimb.2022.881071 (PMC9247299; doi:10.3389/fcimb.2022.881071)

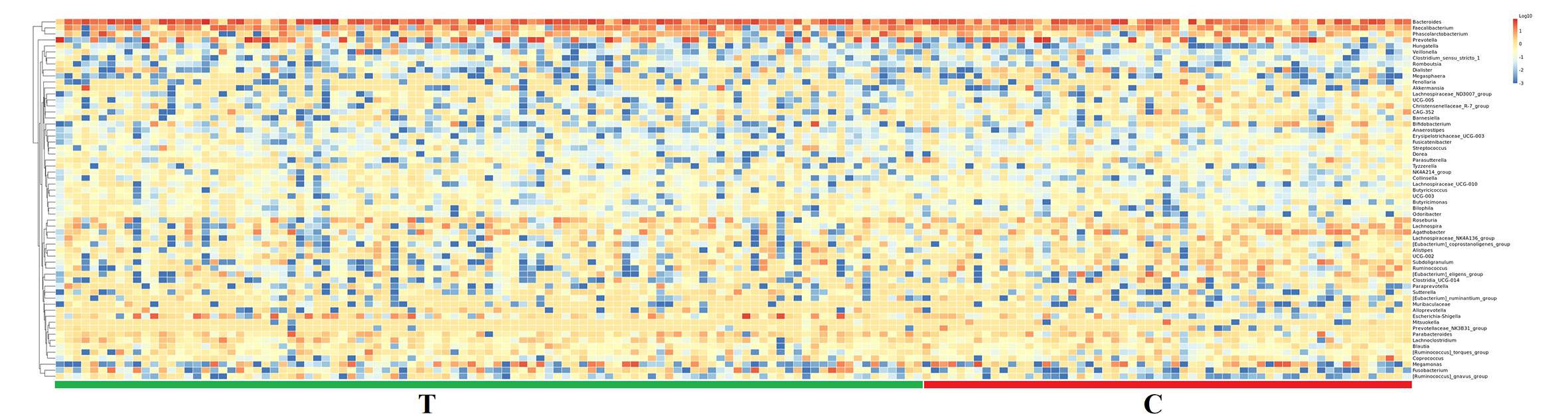

Supplement: Supplementary Figure 1 — Heat map of the relative abundances of the top 60 dominant taxa at genus level in all samples. The genera were displayed from higher abundance (in red) to lower abundance (in blue). [file Image_1.tif]
