# Supplementary material for: Crosstalk Between the Gut and Brain: Importance of the Fecal Microbiota in Patient With Brain Tumors
Source: Front Cell Infect Microbiol. 2022 Jun 17;12:881071. doi: 10.3389/fcimb.2022.881071 (PMC9247299; doi:10.3389/fcimb.2022.881071)
Supplement: Supplementary file 2 [file Table_1.docx]

| Family | mean_T | mean_C | sd_T | sd_C | Wilcoxon.test |
| --- | --- | --- | --- | --- | --- |
| Bifidobacteriaceae | 0.941188119 | 1.627614035 | 3.708280957 | 2.371165384 | 0.000113429 |
| Bacteroidaceae | 27.37923762 | 21.07492982 | 17.67254227 | 15.30036998 | 0.026824294 |
| Barnesiellaceae | 0.265306931 | 0.414982456 | 0.65827626 | 0.618614364 | 0.036883648 |
| RF39 | 0.064316832 | 0.353263158 | 0.242594309 | 0.919415916 | 0.000236384 |
| Christensenellaceae | 0.269079208 | 0.748929825 | 0.811126028 | 1.182428356 | 0.000219788 |
| Clostridia_UCG-014 | 0.258059406 | 0.947561404 | 0.887523688 | 2.421156284 | 0.004007576 |
| Lachnospiraceae | 17.08278218 | 22.306 | 9.227308776 | 10.91439575 | 0.001835571 |
| Monoglobaceae | 0.107871287 | 0.264894737 | 0.194621307 | 0.386847639 | 0.00162495 |
| Ruminococcaceae | 10.42837624 | 13.19657895 | 8.556310664 | 8.031859145 | 0.028224415 |
| Tissierellales | 0.06450495 | 0.958894737 | 0.472368979 | 3.872286528 | 0.000483139 |
| Fusobacteriaceae | 2.126663366 | 0.207403509 | 4.880515787 | 0.633055088 | 3.84E-06 |
| Enterobacteriaceae | 6.216643564 | 1.48745614 | 9.963726756 | 2.698279148 | 1.04E-06 |
| Akkermansiaceae | 0.794891089 | 1.066649123 | 4.168368765 | 3.920094785 | 0.042486507 |

**Supplementary Table 1. The comparison of relative abundant at family level in microbiome between brain tumours group and healthy controls**
