# Supplementary material for: Crosstalk Between the Gut and Brain: Importance of the Fecal Microbiota in Patient With Brain Tumors
Source: Front Cell Infect Microbiol. 2022 Jun 17;12:881071. doi: 10.3389/fcimb.2022.881071 (PMC9247299; doi:10.3389/fcimb.2022.881071)
Supplement: Supplementary file 3 [file Table_2.docx]

| **Genus** | mean_T | mean_C | sd_T | sd_C | Wilcoxon.test |
| --- | --- | --- | --- | --- | --- |
| Bifidobacterium | 0.940287129 | 1.550175439 | 3.70830312 | 2.390206119 | 0.001062518 |
| Bacteroides | 27.37923762 | 21.07492982 | 17.67254227 | 15.30036998 | 0.026824294 |
| RF39 | 0.064316832 | 0.353263158 | 0.242594309 | 0.919415916 | 0.000236384 |
| Christensenellaceae_R-7_group | 0.261059406 | 0.733684211 | 0.806609556 | 1.169478152 | 0.000229216 |
| Clostridia_UCG-014 | 0.258059406 | 0.947561404 | 0.887523688 | 2.421156284 | 0.004007576 |
| Agathobacter | 1.849663366 | 3.602754386 | 3.440018443 | 4.402492096 | 0.001097551 |
| Anaerostipes | 0.087277228 | 0.339561404 | 0.205542264 | 1.123225601 | 0.000288734 |
| Fusicatenibacter | 0.190158416 | 0.557824561 | 0.336600913 | 0.9148642 | 0.000854573 |
| Hungatella | 0.364554455 | 0.018578947 | 1.26443394 | 0.043620911 | 0.000911661 |
| Lachnospira | 1.404980198 | 2.292508772 | 2.179406057 | 2.208302379 | 0.000174728 |
| Lachnospiraceae_ND3007_group | 0.183306931 | 0.708526316 | 0.30741154 | 1.142464646 | 0.000479881 |
| Lachnospiraceae_NK4A136_group | 0.718128713 | 1.201298246 | 1.446822772 | 1.827317286 | 0.007635779 |
| Lachnospiraceae_UCG-004 | 0.133089109 | 0.248859649 | 0.186316564 | 0.308448562 | 0.000718485 |
| [Eubacterium]_eligens_group | 0.685960396 | 2.44677193 | 1.357792767 | 4.365811788 | 5.76E-05 |
| [Eubacterium]_ruminantium_group | 0.299663366 | 0.563631579 | 0.739496549 | 0.936594832 | 0.010547839 |
| [Eubacterium]_ventriosum_group | 0.099118812 | 0.306561404 | 0.332487813 | 0.99217951 | 0.033084712 |
| [Ruminococcus]_gnavus_group | 0.574514851 | 0.154859649 | 1.255631065 | 0.539207336 | 7.11E-07 |
| Monoglobus | 0.107871287 | 0.264894737 | 0.194621307 | 0.386847639 | 0.00162495 |
| Flavonifractor | 0.151277228 | 0.082614035 | 0.216647323 | 0.162645183 | 0.002844182 |
| NK4A214_group | 0.149821782 | 0.334245614 | 0.264463094 | 0.665340012 | 0.0146964 |
| UCG-002 | 0.610435644 | 0.932719298 | 1.035599164 | 1.233580311 | 0.022869552 |
| UCG-005 | 0.320950495 | 0.665122807 | 0.573431711 | 1.444890926 | 0.035539546 |
| CAG-352 | 0.443514851 | 0.843526316 | 1.251438665 | 1.648649903 | 0.001142252 |
| Ruminococcus | 0.74370297 | 1.505473684 | 1.467734837 | 2.779365062 | 0.017100469 |
| Subdoligranulum | 0.947732673 | 2.252894737 | 1.804993556 | 2.929387897 | 9.93E-06 |
| Fenollaria | 0.010633663 | 0.46854386 | 0.079534737 | 2.145203662 | 0.009124532 |
| Fusobacterium | 2.126663366 | 0.154017544 | 4.880515787 | 0.511193194 | 6.50E-07 |
| Parasutterella | 0.516960396 | 1.433508772 | 0.842073583 | 2.219069344 | 3.26E-05 |
| Sutterella | 0.888623762 | 0.798912281 | 1.267258552 | 2.241207537 | 0.046859483 |
| Escherichia/Shigella | 4.076841584 | 0.905298246 | 8.198203575 | 1.624522963 | 7.57E-05 |
| Akkermansia | 0.794891089 | 1.066649123 | 4.168368765 | 3.920094785 | 0.042486507 |

**Supplementary Table 2. The comparison of relative abundant at genus level in microbiome between brain tumours group and healthy controls**
